# Preparation, Characterization, and Antibacterial Properties of SA/PVA/RNC/ZnONPs Composite Hydrogels

**DOI:** 10.3390/biology15211986

**Published:** 2026-11-02

**Authors:** Guanghua Yang, Heling Yu, Zhiqiang Cai

**Affiliations:** School of Pharmacy & School of Biological and Food Engineering, Changzhou University, Changzhou 213164, China; yguanghua@cczu.edu.cn (G.Y.);

**Keywords:** sodium alginate, poly(vinyl alcohol), rape-straw-derived cellulose, zinc oxide, composite gel beads, antibacterial activity

## Abstract

SA/PVA/RNC/ZnONPs composite gel beads were prepared by calcium-mediated ionic cross-linking and freeze-drying, using sodium alginate (SA), poly(vinyl alcohol) (PVA), rape-straw-derived cellulose material (RNC) and zinc oxide particles (ZnONPs). SEM, FTIR, and XRD were used to examine the dried composite morphology, functional groups, and crystalline features. Swelling, water loss and antibacterial performance were evaluated. Among the tested formulations, 0.8 wt% PVA gave the highest reported equilibrium swelling ratio, and 1.0 wt% ZnONPs was selected on the basis of antibacterial screening. The antibacterial response against *Escherichia coli*, *Pseudomonas aeruginosa*, and *Staphylococcus aureus* depended on material dosage and contact time. Antibacterial function was retained during repeated use and storage, and activity after pH preconditioning was lower at pH 1 than at pH 2–9. These observations do not establish nanoparticle immobilization, mechanical durability, or application safety. RNC loading and cross-linking conditions were fixed, so the selected formulation is not a globally optimized composition. Particle dimensions, colloidal behavior, hydrated-state mechanics, zinc release, ROS generation, and cytocompatibility require direct measurements. The material therefore represents a candidate for further investigation as an antibacterial composite, with proposed mechanisms and application suitability remaining to be validated.

## Simple Summary

SA/PVA/RNC/ZnONPs composite gel beads were prepared and its antibacterial response against *Escherichia coli*, *Pseudomonas aeruginosa*, and *Staphylococcus aureus* depended on material dosage and contact time. Particle dimensions, colloidal behavior, hydrated-state mechanics, zinc release, ROS generation, and cytocompatibility were also investigated.

## 1. Introduction

Nanocomposite hydrogel dressings have been investigated for bacterial infection management, including photothermal treatment combined with controlled antibiotic release [1]. Metal-nanoparticle/plant-extract composites and silver–ginger composites in natural hydrogel matrices provide further examples of antimicrobial material design [2,3]. Reviews describe hydrogel classifications, network properties and biomedical applications [4,5], while natural-polymer nanocomposite reviews discuss functional fillers for wound-related materials [6,7]. These studies motivate composite design but do not establish the safety or efficacy of the present formulation.

Sodium alginate (SA) is an anionic polysaccharide that forms calcium-cross-linked networks, as discussed in alginate biomaterial reviews [8,9,10]. Broader network-design context includes collagen/guar-gum semi-interpenetrating hydrogels [11] and three-dimensional polymer networks [12]. ZnO-containing gelatin hydrogels provide a relevant example of antibacterial filler incorporation [13]. Cerium oxide antibacterial materials involve different oxide chemistry [14], while nanoparticle surface-functionalization research highlights dispersion as a separate design consideration [15]. Broader hydrogel applications [16], alginate/gelatin/PVA networks [17], and alginate microparticles embedded in PVA [18] support consideration of blended matrices; they do not directly demonstrate cellulose reinforcement in this system.

Plant-assisted ZnO synthesis has been reported using saffron extract [19], whereas agricultural-waste nanocellulose preparation and packaging applications are reviewed by Ren et al. [20]. These sources provide general preparation context; the saffron study is not a dandelion-specific synthesis protocol, and the review is not evidence that the present RNC has nanoscale dimensions.

The supplied reference list also includes carbon-fiber/PLA–PHA composites [21], polyester/glass/wool composites [22], and copper-matrix composites [23]. These provide examples of structure–property analysis in other material classes, rather than validation of hydrogel-specific SEM, FTIR, or XRD assignments. More directly related examples include CNC-containing multifunctional hydrogels [24] and nanocellulose aerogels for water disinfection [25]. A review of graphene-containing biodegradable antibacterial composites offers additional, chemically distinct context [26]. Alginate-modified geopolymer concrete [27] and in situ SEM of CaCO_3_ calcination/CaO densification [28] concern different processes and cannot substantiate antibacterial stability or dried hydrogel morphology.

Other architectures in the reference list include porous polymer blends for electromagnetic shielding [29], PVA/graphene oxide/silver packaging composites [30] and PVA/CNF phase-change aerogels [31]. These comparisons illustrate different filler and pore-design objectives, not equivalent antibacterial mechanisms. ZnS/ZnO@g-C_3_N_4_ photocatalytic heterojunctions [32], ZnO/nanocellulose-functionalized electrospun membranes [33], PANI/ZnO composites [34], ZnO/chitosan/CMC photocatalytic adsorbents [35] and fiber/filler-reinforced epoxy composites [36] likewise differ in matrix, illumination, or intended use. Their findings should not be transferred quantitatively to the present calcium-cross-linked beads.

SA was selected as the calcium-responsive bead-forming matrix; PVA as a hydrophilic blending polymer; RNC as an agricultural-residue-derived hydroxyl-rich filler; and ZnO as the antibacterial component. These complementary roles motivate the composition but do not demonstrate synergy or safety. S/P/C denotes the ternary polymer–cellulose matrix, whereas S/P/C/ZnONPs contains four components. Here, RNC denotes the rape-straw-derived cellulose material obtained by chemical treatment and ball milling; nanoscale dimensions and cellulose-nanocrystal identity remain unverified. This study evaluates the morphology, functional-group features, crystalline features, swelling behavior and antibacterial activity of SA/PVA/RNC/ZnONPs composite gel beads. RNC loading and calcium chloride cross-linking conditions were held constant while selected formulation variables were screened. The study does not establish a globally optimized network or demonstrate mechanical strengthening, specific antibacterial pathways, or safety for biomedical and food-contact use. These evidence gaps are discussed together with the measurements needed to address them.

## 2. Results and Discussion

### 2.1. Characterization and Analysis of Composite Materials

#### 2.1.1. Scanning Electron Microscopy (SEM) Results and Analysis

**Figure 1:**
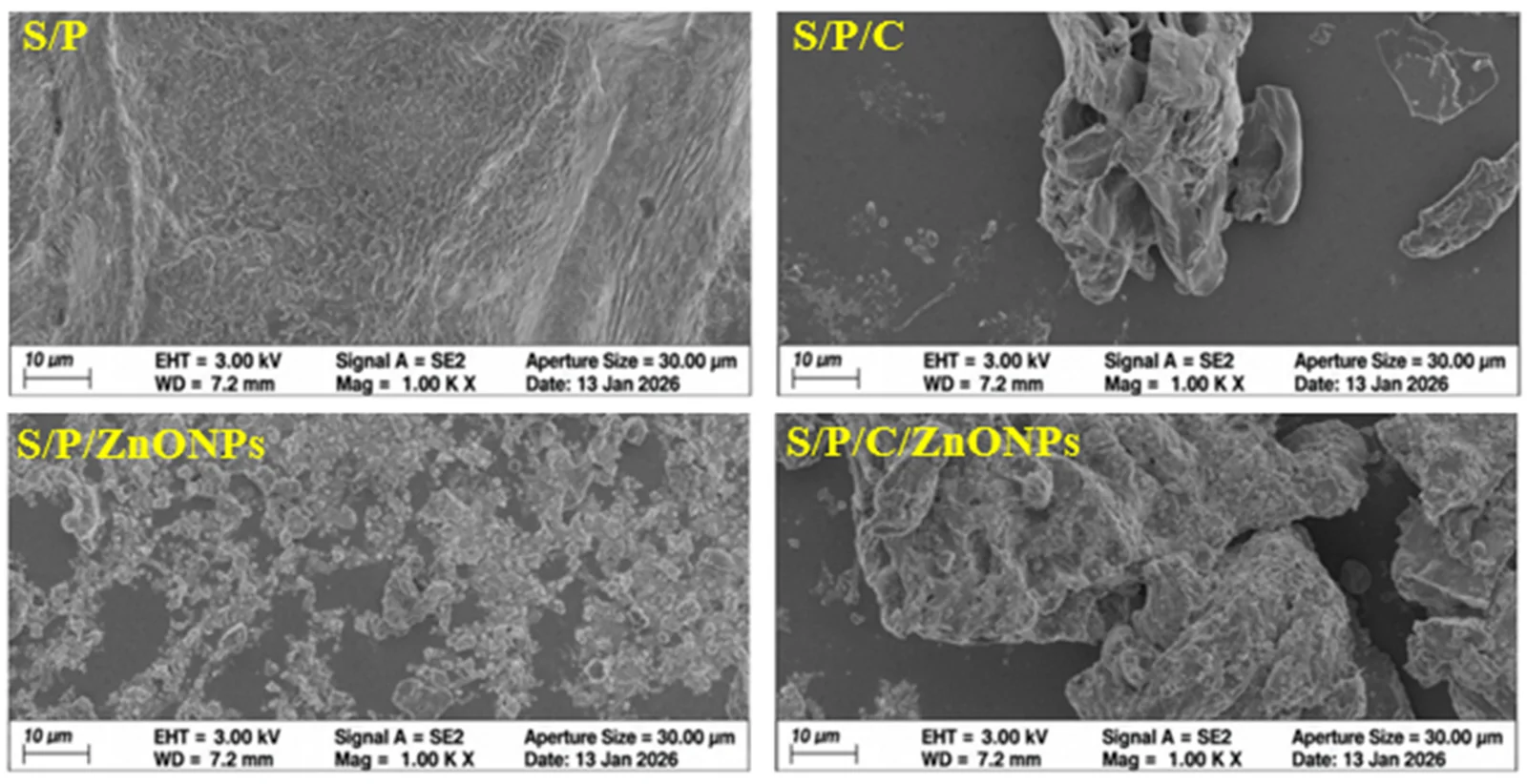
SEM images of S/P hydrogel, S/P/C hydrogel, S/P/ZnONPs hydrogel, and S/P/C/ZnONPs hydrogel.

SEM was used to compare the dried composite morphologies in Figure 1. The S/P matrix exhibited a fibrous network, whereas cellulose-containing S/P/C samples showed more compact, irregular aggregates with visible voids. ZnONPs-containing formulations displayed particulate surface features. The four-component S/P/C/ZnONPs material retained an aggregate-like structure with a rougher surface. These observations describe dried morphology and do not establish uniform ZnO dispersion throughout the hydrated composite. Hydrogen bonding among hydroxyl-rich RNC and PVA and the oxygen-containing groups of SA, together with possible ZnO–polymer surface interactions, may help associate the components. Physical confinement within the calcium-cross-linked matrix could also limit particle mobility. These are proposed explanations, not demonstrated stabilization mechanisms. No TEM, DLS, zeta-potential or quantitative image analysis was performed to show that RNC prevented ZnONPs aggregation. Surface charge and electrostatic stabilization therefore cannot be assigned. Dispersion homogeneity across independently prepared batches was not evaluated.

#### 2.1.2. Fourier Transform Infrared Spectroscopy (FTIR) Analysis

**Figure 2:**
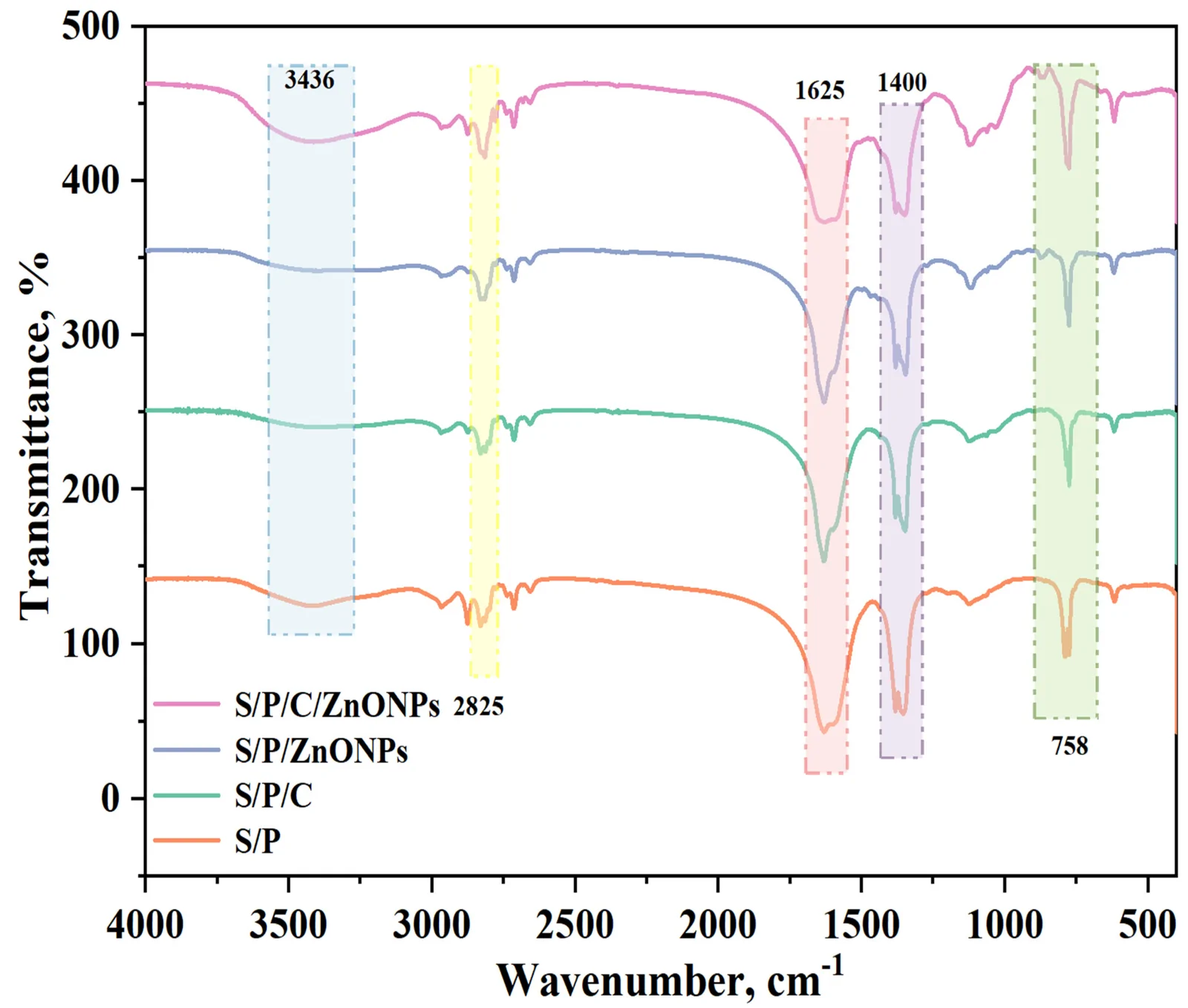
FTIR spectra of S/P hydrogel, S/P/C hydrogel, S/P/ZnONPs hydrogel, and S/P/C/ZnONPs hydrogel.

FTIR spectra were used to compare the functional-group features of the formulations (Figure 2). The broad band near 3436 cm^−1^ is consistent with hydroxyl stretching. In the SA/PVA matrix, the bands around 1625 and 1400 cm^−1^ are more appropriately discussed in relation to alginate carboxylate vibrations, with possible overlap from absorbed water near 1625 cm^−1^, rather than assigned to amide I and II without an identified amide-containing component. Changes in these bands after filler addition may indicate altered local hydrogen-bonding environments. The signal reported near 758 cm^−1^ should not, by itself, be treated as definitive evidence of Zn–O bonding or coordination to the polymer matrix; comparison with isolated-component spectra is needed. FTIR does not directly establish electrostatic stabilization, suppressed aggregation, or mechanical enhancement.

#### 2.1.3. X-Ray Diffraction (XRD) Analysis

**Figure 3:**
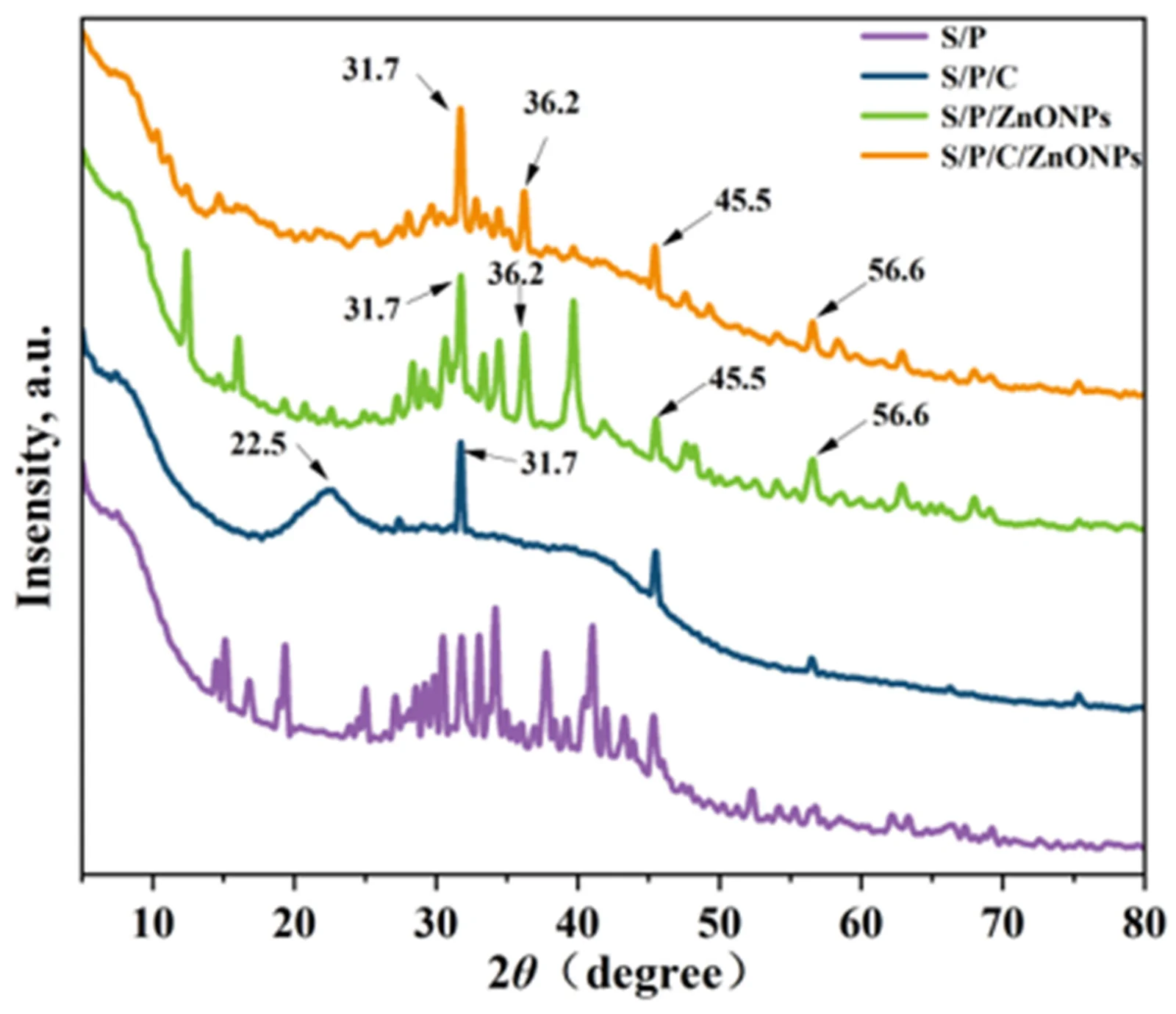
XRD patterns of S/P hydrogel, S/P/C hydrogel, S/P/ZnONPs hydrogel, and S/P/C/ZnONPs hydrogel.

XRD patterns of the composite formulations are shown in Figure 3. The S/P matrix was predominantly amorphous, and the cellulose-containing sample showed a weak feature near 22.5°. ZnONPs-containing samples exhibited additional crystalline reflections. Identification of each ZnO reflection requires comparison with a reference diffraction pattern; the assignment of the feature reported near 45.5° should be checked against the original diffractogram before indexing. Differences in peak intensity and width may reflect loading, crystallite characteristics, preferred orientation, or specimen preparation. They do not independently demonstrate improved dispersion, enhanced mechanical stability, or exclusively physical interactions. The available XRD patterns do not establish the primary-particle dimensions or aggregation state of ZnONPs or RNC. The displayed S/P control also contains sharp reflections, so its phase constitution cannot be described as wholly amorphous or used to assign every shared reflection to added ZnO. The feature near 22.5° may include overlapping cellulose and polymer scattering and is not sufficient to determine the cellulose polymorph. The peak marked 46.2° must remain unassigned until the original scan and calibration are checked; it should not be relabeled as a ZnO reflection solely to match an expected pattern. Phase purity has not been established.

### 2.2. Effect of PVA Content on Swelling Performance and Water Loss Rate of Aerogels

**Figure 4:**
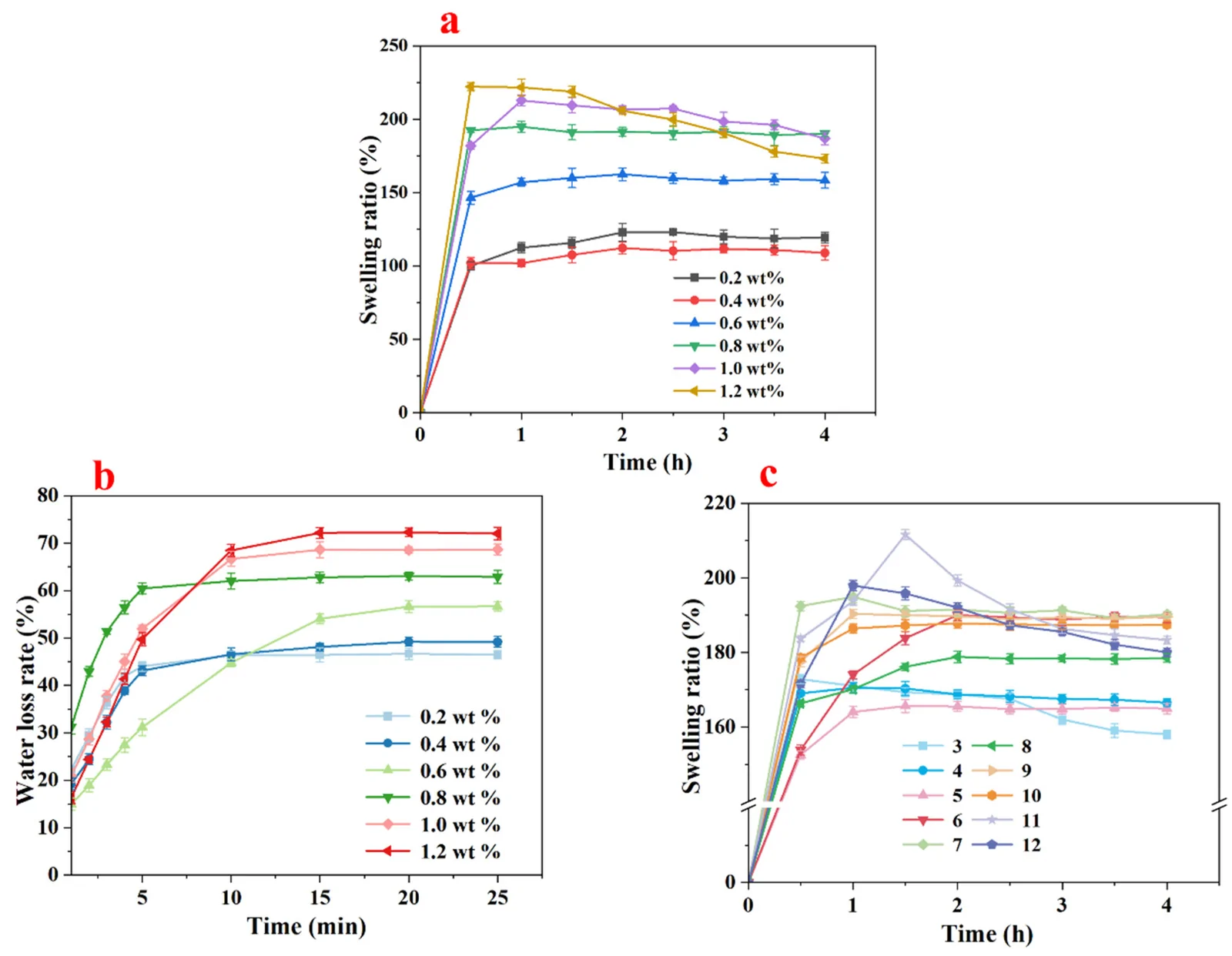
Effect of different PVA loadings (0.2, 0.4, 0.6, 0.8, 1.0, and 1.2 wt%) on the swelling ratio (**a**) and water loss rate (**b**) of S/P/C/ZnONPs hydrogels; and the swelling ratio of the hydrogel in solutions with different pH values (**c**).

As shown in Figure 4a, formulations containing 0.2–0.8 wt% PVA reached the reported swelling equilibrium within 1 h. The equilibrium swelling ratio increased to approximately 195% at 0.8 wt% PVA and decreased at higher PVA contents. Changes in polymer hydration and network organization may contribute to this response [37,38], but partial dissolution was not quantified and cannot be identified as the established cause. In Figure 4b, the water loss rate increased over time and was lowest for the 0.2 wt% PVA formulation under the tested conditions. This observation describes water retention in the specified assay and does not measure mechanical strength or cross-link density. Figure 4c shows swelling ratios of approximately 160–195% over pH 4–10, with lower swelling at pH 3 and pH 11–12. Protonation of alginate carboxyl groups may contribute to acidic contraction, while changes in ionic interactions or polymer integrity may contribute under alkaline conditions [39]. Neither mechanism was measured directly. Swelling alone cannot distinguish reversible contraction from erosion, dissolution, or loss of cross-links. The selection of 0.8 wt% PVA therefore applies to swelling within the tested formulation range, rather than to overall hydrogel performance.

The visible voids in the freeze-dried specimens are qualitatively compatible with water-access pathways during rehydration, while hydroxyl and carboxyl-containing components provide hydration sites. Accessible connected pores may facilitate uptake, whereas polymer association can restrict expansion. Water retention additionally depends on pore connectivity, capillary confinement, polymer–water interactions and the evaporation conditions. Thus, high swelling need not imply low water loss: 0.8 wt% PVA gave the highest reported swelling, whereas 0.2 wt% gave the lowest water loss in the 60 °C assay. No quantitative pore-size distribution, porosity or hydrated-state imaging was obtained, and SEM of dried specimens cannot establish the hydrated pore architecture or a causal pore–swelling relationship. Matched porosity measurements, cryo-SEM or other hydrated-state imaging, swelling kinetics, and controlled-temperature/humidity desorption tests are proposed to test this relationship.

Published antibacterial matrices include silver-bearing chitosan films [40] and chitin/ZnO hydrogels [41]. Ionic-liquid-stabilized MXene/chitosan hydrogels concern a different filler-stabilization problem [42], whereas cellulose-surface carboxyl groups have been examined in ZnO/cellulose interfaces [43]. Cellulose/chitosan/zinc-ion hydrogels [44], polysaccharide/Zn^2+^ physical networks [45] and gelatin particles containing ZnO and silver [46] provide further examples of zinc-containing materials. Other hydrogel systems employ slightly acidic electrolyzed water [47] or multifunctional dressings [48]. These studies contextualize antibacterial matrix design but do not establish RNC-mediated ZnO dispersion or synergy in the present beads.

### 2.3. Effect of Composite Gels on Antibacterial Performance

**Figure 5:**
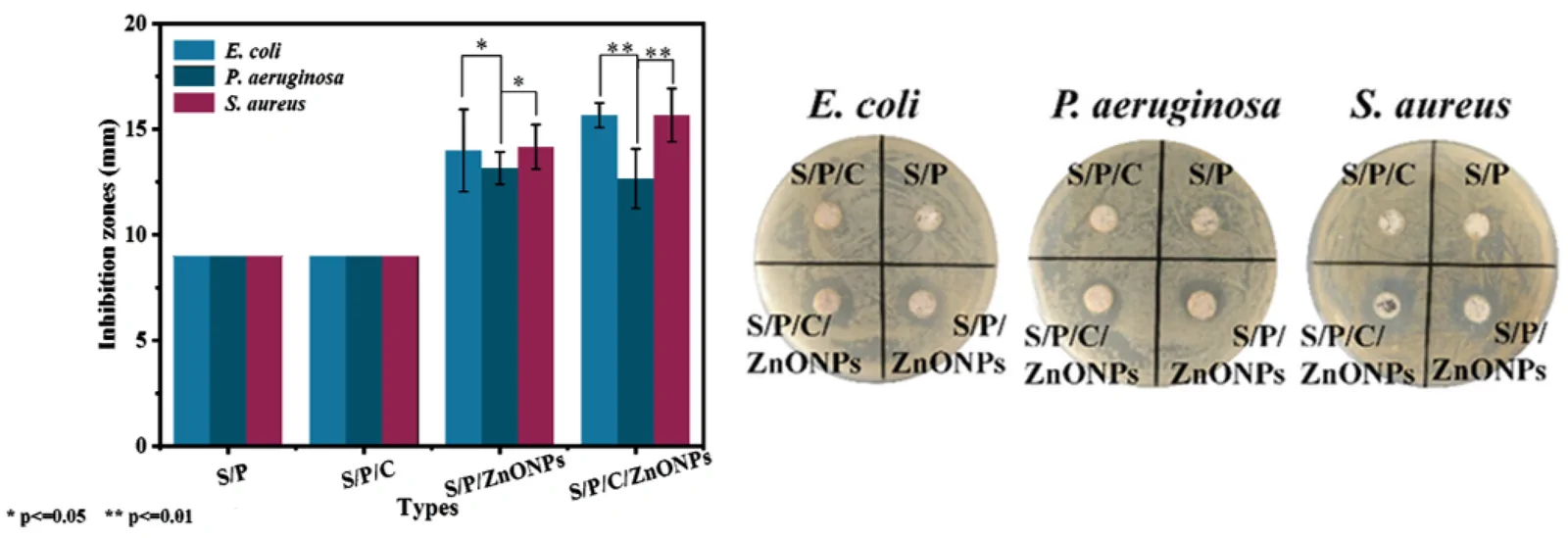
Antibacterial images of S/P hydrogel, S/P/C hydrogel, S/P/ZnONPs hydrogel, and S/P/C/ZnONPs hydrogel.

The agar well diffusion assay compared antibacterial responses against *E. coli*, *P. aeruginosa*, and *S. aureus* (Figure 5). The S/P and S/P/C samples gave diameters of 9 mm, corresponding to the well diameter and indicating no visible inhibition halo beyond the well. The reported diameters for S/P/ZnONPs were approximately 14.0, 13.2, and 14.2 mm, respectively, compared with 15.7, 12.7, and 15.7 mm for S/P/C/ZnONPs. Thus, the RNC-containing formulation produced larger reported zones for *E. coli* and *S. aureus*, but not for *P. aeruginosa*. Without an appropriate statistical comparison, these differences should not be described as significant or as evidence of universal synergy. Differences in diffusion and accessibility of antibacterial species may contribute. Suppression of particle aggregation, sustained Zn^2+^ release, ROS generation, and enhanced bacterial capture were not quantified in this work and remain possible explanations rather than demonstrated mechanisms.

### 2.4. Effect of ZnONPs Dosage on Antibacterial Performance

**Figure 6:**
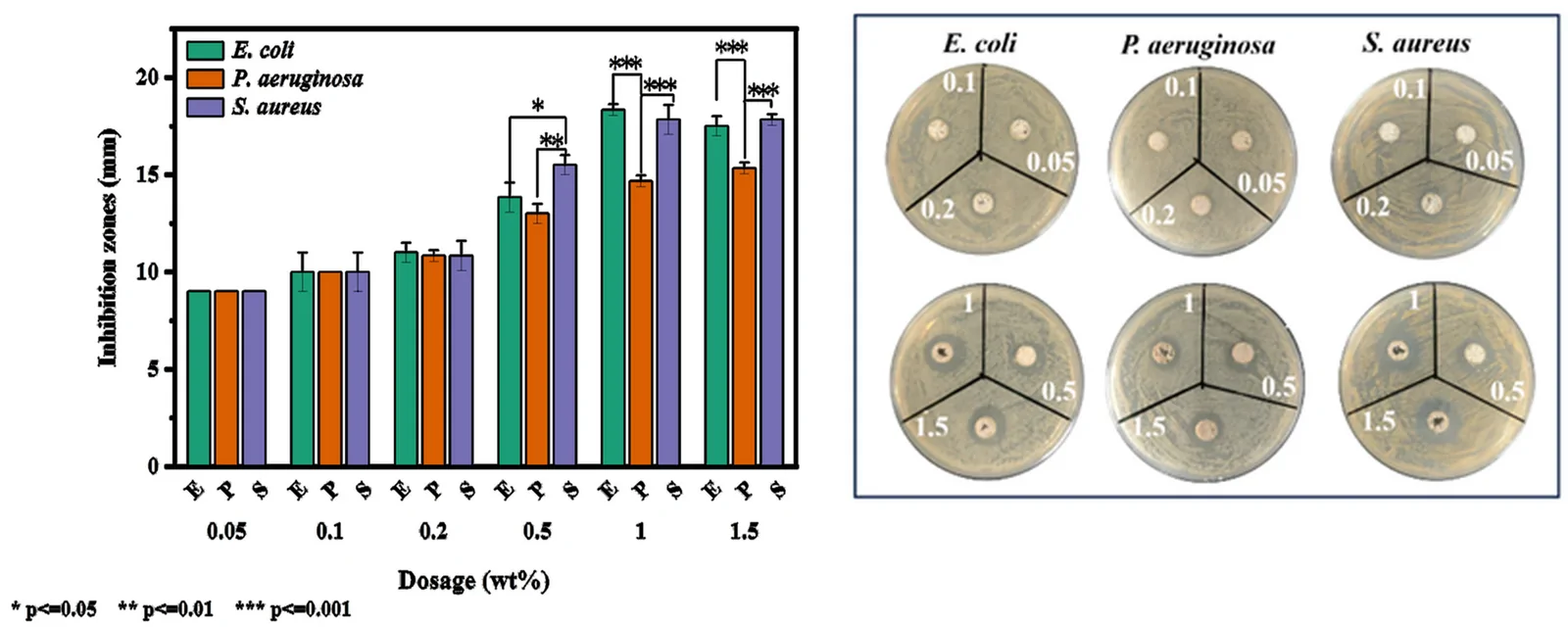
Effect of different ZnONPs contents on the antibacterial activity of S/P/C/ZnONPs hydrogel.

Figure 6 compares ZnONPs mass fractions of 0.05, 0.1, 0.2, 0.5, 1.0, and 1.5 wt% in the composite formulation. The reported inhibition zones increased up to 1.0 wt% ZnONPs, reaching approximately 18.2, 14.2, and 17.5 mm for *E. coli*, *P. aeruginosa*, and *S. aureus*, respectively, and decreased at 1.5 wt%. Accordingly, 1.0 wt% was selected within this screening range. This selection does not establish an overall optimum because RNC loading, CaCl_2_ concentration, and cross-linking time were not varied, and release behavior, mechanical properties, and cytotoxicity were not included in the selection criteria. The decrease at 1.5 wt% could reflect altered aggregation, diffusion, or particle accessibility, but none was measured. The antibacterial screening selected a nominal composition rather than demonstrating the lowest effective zinc dose or an efficacy–safety optimum. PVA and ZnO were not jointly varied in a factorial experiment.

### 2.5. Effect of Composite Gel Dosage and Contact Time on Antibacterial Performance

**Figure 7:**
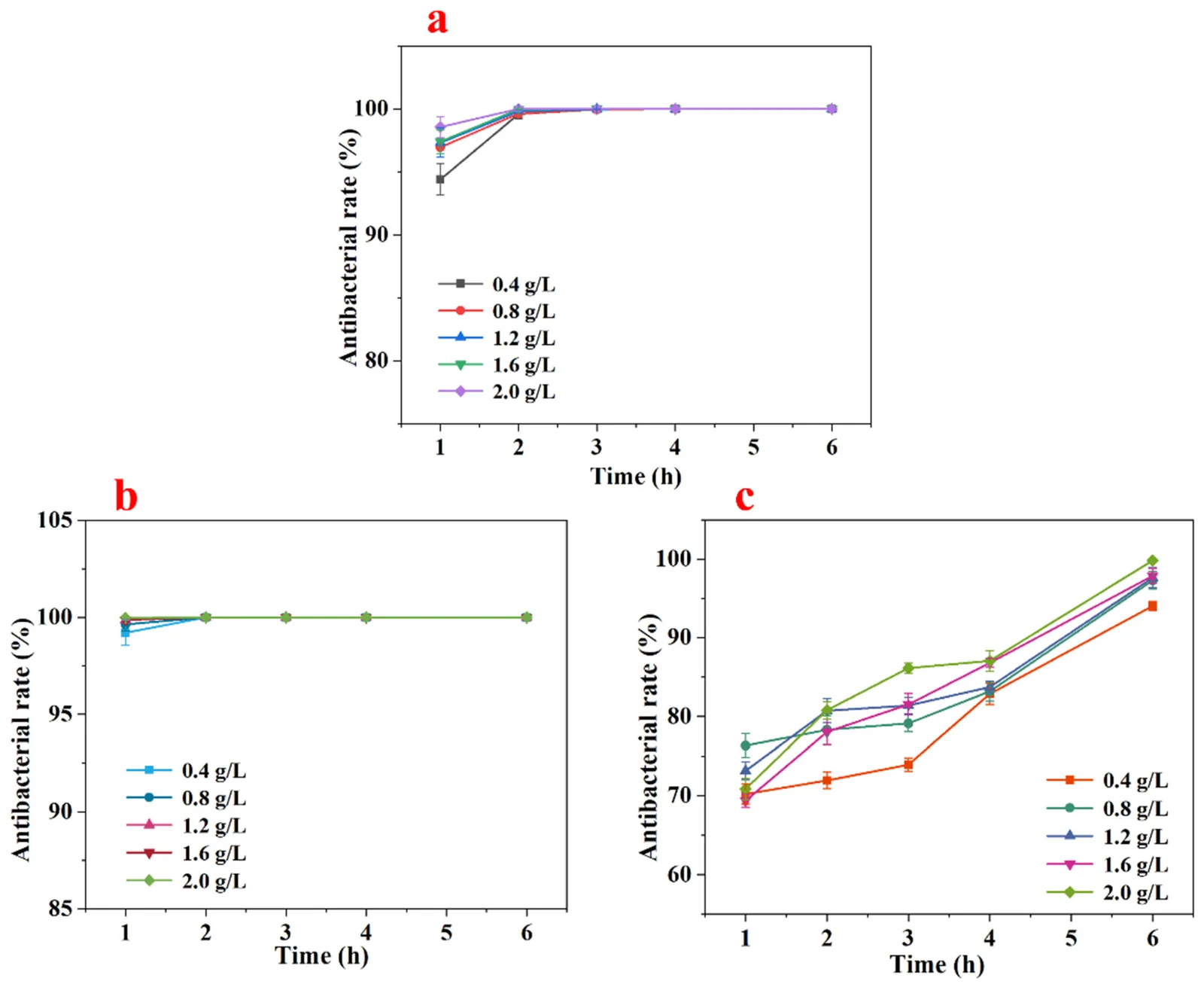
Antibacterial performance of S/P/C/ZnONPs hydrogel against *Escherichia coli* (**a**), *Pseudomonas aeruginosa* (**b**), and *Staphylococcus aureus* (**c**) at different dosages (0.4, 0.8, 1.2, 1.6, 2.0 g/L) and contact times (1.0, 2.0, 3.0, 4.0, 6.0 h).

Figure 7 shows the variation in antibacterial rates of the S/P/C/ZnONPs (1.0%) composite gel against *E. coli* (a), *P. aeruginosa* (b), and *S. aureus* (c) at different dosages (0.4, 0.8, 1.2, 1.6, 2.0 g/L) and contact time (1.0, 2.0, 3.0, 4.0, 6.0 h). Overall, the antibacterial rates against all three strains increased with increasing gel dosage and prolonged contact time, showing obvious dose-dependent and time-dependent behaviors. Under low dosage (0.4 g/L) and short contact time (1 h), the antibacterial rates were relatively moderate, reaching approximately 94.4%, 99.2%, and 70.2% for *E. coli*, *P. aeruginosa*, and *S. aureus*, respectively, all exhibiting favorable antibacterial effects. Reported values reached 100% for *E. coli* and *P. aeruginosa* at relatively low dosages and short durations. At a dosage of 0.4 g/L, a reported antibacterial rate of 100% against *E. coli* and *P. aeruginosa* was obtained within 2 h. At a dosage of 2.0 g/L, nearly 100% inhibition of S. aureus was achieved within 6 h. In addition, when the dosage reached 1.6 g/L and above, the antibacterial rate remained at a high level at all time points, indicating that the S/P/C/ZnONPs gel possesses highly efficient and broad-spectrum antibacterial performance, and can exert rapid and stable antibacterial effects at relatively high dosages.

### 2.6. Reusability and Stability of Composite Aerogels

**Figure 8:**
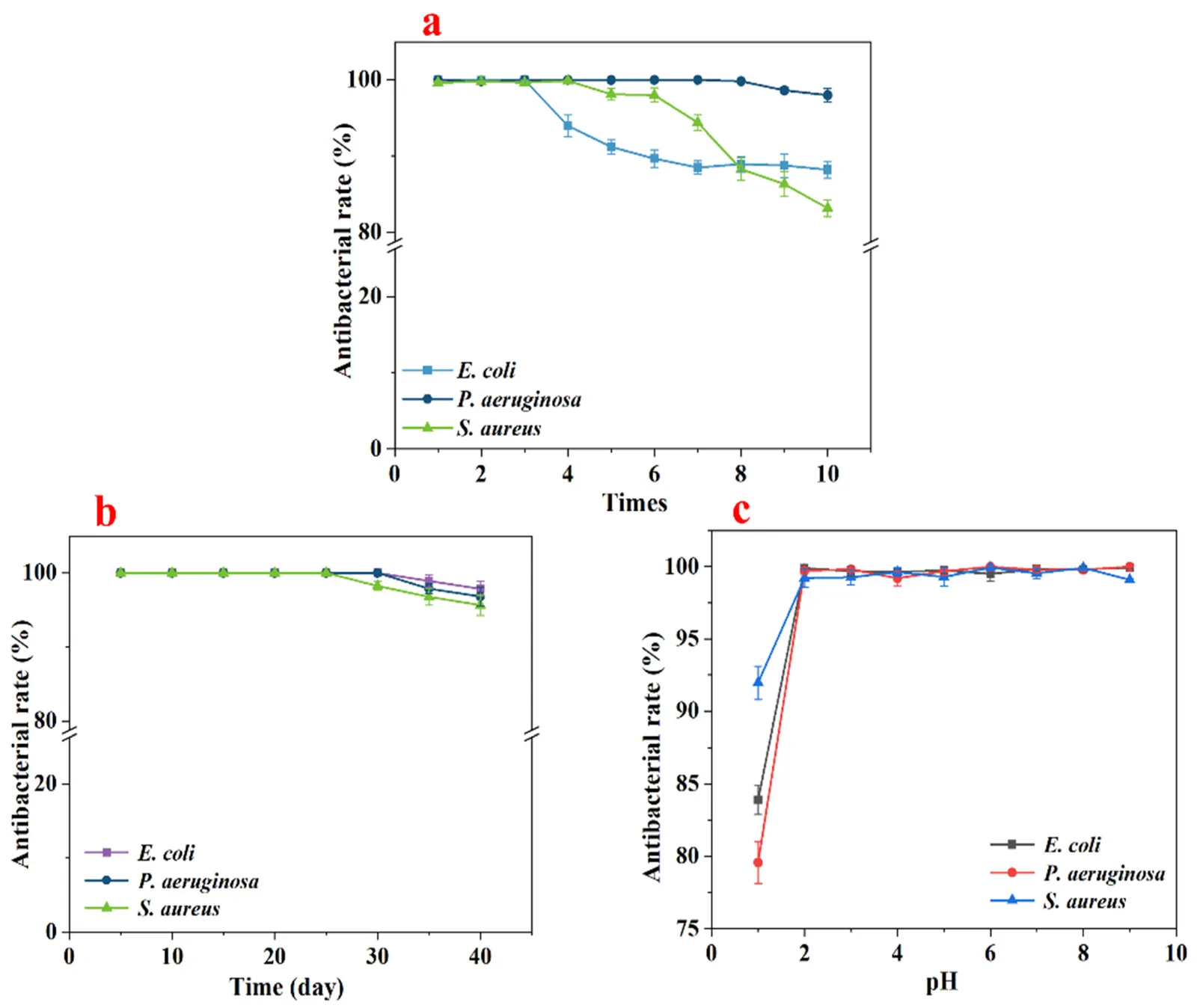
Repeated antibacterial performance of S/P/C/ZnONPs hydrogel (1, 2, 3, 4, 5, 6, 7, 8, 9, and 10 cycles) (**a**); antibacterial images after different storage days (5, 10, 15, 20, 25, 30, 35, and 40 days) (**b**); antibacterial activity after immersion in solutions with different pH values (1–9) (**c**).

Figure 8a reports antibacterial activity over repeated use. *P. aeruginosa* inhibition remained above 97% over the ten cycles shown, whereas the reported values for *E. coli* and *S. aureus* declined to approximately 88% and 83% at cycle 10. These data indicate retention of antibacterial function under the reported assay conditions, but do not establish preservation of the network or Zn content. Loss of zinc-containing species, matrix erosion, fouling, or changes caused by rinsing and drying are possible explanations for declining activity; their relative contributions were not measured. Figure 8b shows storage-related antibacterial activity: values were reported as 100% during days 5–25, and at day 40 were approximately 95% for S. aureus and above 96% for *E. coli* and *P. aeruginosa*. These observations do not demonstrate absence of aggregation or leaching and do not establish a practical shelf life. Figure 8c shows antibacterial activity after pH preconditioning and rinsing. Reported inhibition exceeded 99% after treatment at pH 2–9, but fell to approximately 84%, 80%, and 92% for *E. coli*, *P. aeruginosa*, and *S. aureus*, respectively, after pH 1 treatment. This protocol assesses retained activity after conditioning, not antibacterial efficacy during continuous exposure at each pH. Acid-promoted ZnO dissolution could increase zinc loss during preconditioning and rinsing, leaving less zinc for the subsequent assay. Network contraction, erosion, or altered transport could also contribute. Neither zinc release nor morphological changes were measured, so the cause of the activity decline remains unresolved.

Reusability and functional-property comparisons require attention to the assay endpoint: a mechanically strong reusable hydrogel developed for Cu(II)/Pb(II) removal [49] does not validate repeated antibacterial performance. Low-swelling antibacterial alginate [50], CMC/ZnO films [51], CuO/polymer composites [52], and PLGA/ZnO composites evaluated for antibacterial and cytotoxic properties [53] illustrate the need to assess hydration, activity, and biological compatibility separately. Alginate/PVA-containing drug-delivery microspheres [54] and PVA/ZnO controlled-release hydrogels [55] also concern transport in other formulations, rather than proof of sustained zinc release from these beads.

### 2.7. Proposed Antibacterial Pathways and Evidence Limitations

**Figure 9:**
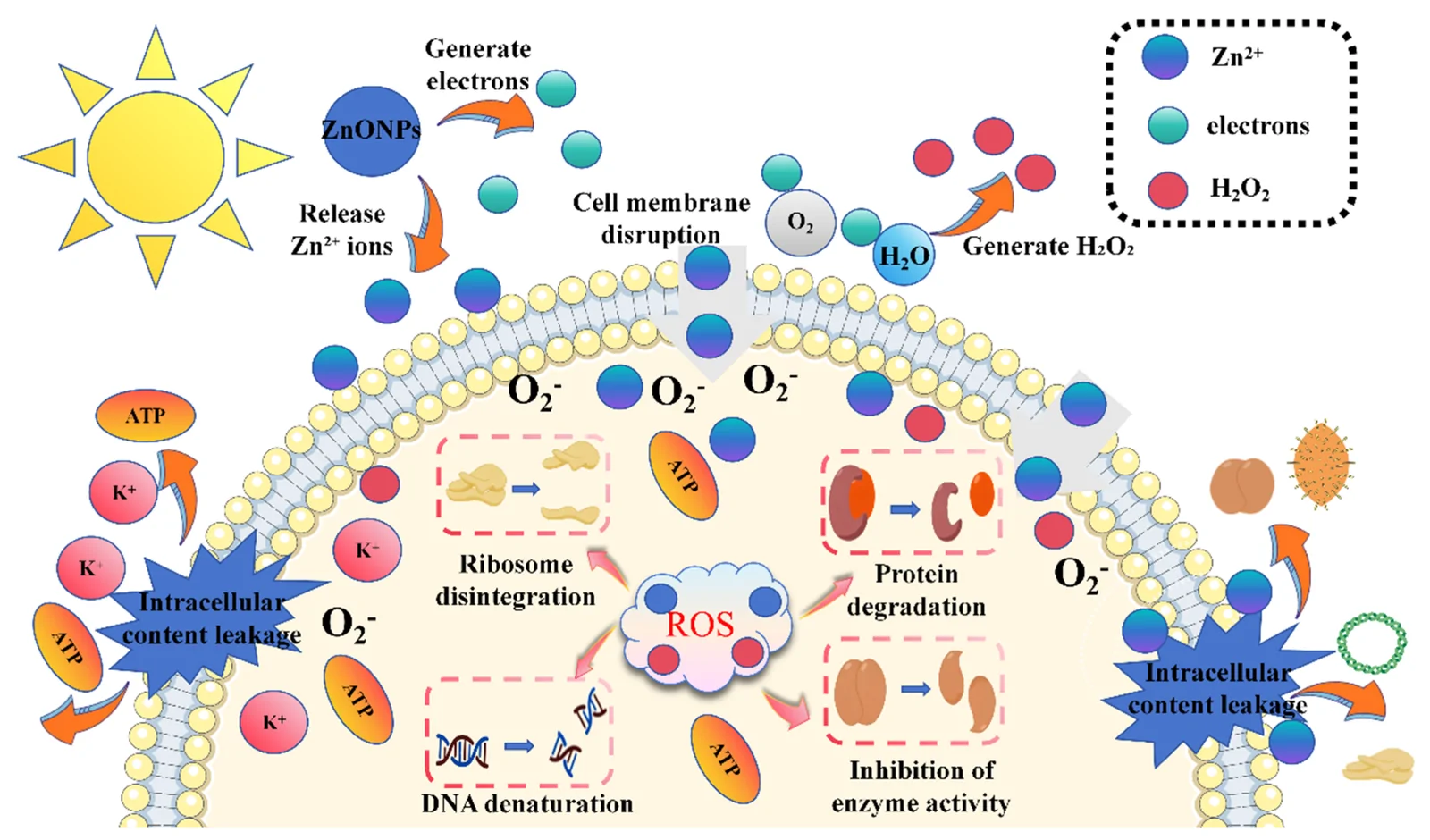
Conceptual antibacterial pathways proposed for the SA/PVA/RNC/ZnONPs composite. Ionic toxicity, oxidative stress, and contact-associated membrane damage are hypotheses; zinc release, ROS production, and bacterial ultrastructural injury were not measured in this study. The arrows do not represent experimentally established causal relationships.

The antibacterial assays establish activity against the tested strains, but do not identify the responsible pathways. Mechanistic studies of ZnO nanoparticles provide relevant hypotheses [56]. A study of sodium hypochlorite against resistant pathogens and biofilms involves a different antimicrobial chemistry [57], and grape-seed-assisted ZnO synthesis concerns a different plant-derived preparation [58]. Neither demonstrates resistance suppression or a specific pathway in this composite. Caron et al. identified soluble zinc as the principal contributor to ZnO-mediated toxicity in Mueller–Hinton broth [59]; that medium-specific result cannot establish the mechanism in the present LB-based assays. Reviews of ZnO antimicrobial mechanisms provide broader context [60]. Figure 9 is therefore a conceptual hypothesis rather than experimental validation of three simultaneous pathways.

Zinc-mediated toxicity is a plausible contribution, but release kinetics, dissolved-zinc concentrations, and zinc speciation were not measured. Time-resolved analysis by ICP-OES or ICP-MS should quantify zinc in the exposure medium, with validated separation of dissolved and particulate fractions, recovery controls, and correction for sampling and medium replacement. A particle-free leachate, a soluble-zinc control matched to measured release, ZnO-free matrix controls, and a contact-separation experiment would help distinguish diffusible and contact-associated effects. Operationally dissolved zinc measured after separation should not automatically be equated with free Zn^2+^ because medium components can bind zinc.

ROS generation and physical membrane damage also remain unverified. Future ROS assays should distinguish cell-free oxidant production from intracellular oxidative responses and include probe-only, material-only, untreated-cell and positive controls, correction for optical interference, and defined illumination conditions. Quantitative peroxide assays or EPR-based radical measurements could complement intracellular fluorescent probes. Scavenger experiments should include controls for direct effects on bacterial growth and zinc chemistry. SEM of identically prepared treated and untreated bacteria, combined with membrane-permeability assays, could assess envelope injury. Shrinkage or rupture in SEM images alone would not demonstrate nanoparticle penetration or mechanical piercing, because chemical injury and preparation artifacts can produce similar appearances. Biological assays that distinguish oxidative responses, membrane injury, and biofilm effects are needed before assigning a mechanism to the present composite.

Accordingly, the present results support antibacterial activity under the tested conditions but do not establish sustained zinc release, ROS-mediated killing, physical membrane piercing, or synergy among these pathways. Resistance development and mammalian-cell compatibility were not examined. Repeated-use antibacterial activity is not a test of resistance development, and activity against bacteria does not establish safety for mammalian cells. The biological evaluation needed for any proposed application is discussed in Section 2.10. Multiple forms of injury could, in principle, make escape through a single altered molecular target less effective than for an agent acting at one target. This is a mechanistic hypothesis, not evidence that this composite reduces resistance emergence. Bacteria may adapt through metal homeostasis, stress responses, or biofilm-associated protection, and prolonged sublethal exposure could provide selection pressure. The material should therefore not be described as non-resistance-inducing. Serial passage of independent bacterial lineages at defined subinhibitory exposures, with unexposed passage controls, standardized susceptibility measurements and post-exposure regrowth tests, is proposed to assess stable susceptibility changes. Comparisons with soluble-zinc and ZnO-free controls and testing after passages without exposure would help distinguish persistent resistance from reversible adaptation. No such experiments were performed here.

RNC may influence antibacterial performance indirectly by changing particle accessibility, local hydration, transport, or bacterial contact, but these contributions remain hypotheses. The inhibition-zone comparison did not show a larger zone for the RNC-containing formulation against every strain, and a single fixed RNC loading cannot establish a dose-dependent contribution or synergy. Cellulose alone should not be assumed to kill bacteria or enhance ROS formation. Cellulose-nanofibril-reinforced wound-dressing hydrogels [61] and chitosan/PVA hydrogels containing Aloe vera and green-synthesized ZnO [62] provide related network-design comparisons. Magnesium-hydroxide-containing multinetwork hydrogels [63] involve a different inorganic component. These examples motivate controlled filler comparisons but do not prove an RNC contribution to antibacterial activity in the present formulation. The hydroxyl-rich RNC surface may provide association sites for polymer chains and ZnO surface groups, while the calcium-alginate network may physically confine zinc-containing domains. Such interactions could reduce particle mobility or redistribute stress but could also promote bridging aggregation when dispersion is poor. Structural stabilization cannot therefore be concluded from filler incorporation alone. A direct comparison of RNC-free and RNC-containing specimens at matched initial Zn content, together with spatial Zn mapping and hydrated cyclic compression, is needed to test whether distribution and mechanical stability improve together.

To test these hypotheses, future work should compare SA/PVA, SA/PVA/RNC, SA/PVA/ZnONPs, and SA/PVA/RNC/ZnONPs at matched specimen geometry and measured initial zinc content, with graded RNC loading. TEM of isolated fillers and quantitative electron-microscopy/elemental maps from multiple predefined surface and cross-sectional fields should assess particle domains, aggregate-area fraction, domain-size distributions and spatial variability. Zn mapping identifies zinc-containing domains but should not automatically be treated as particle-size measurement. A prespecified blinded sampling plan across at least three independently prepared batches would allow within-batch and between-batch variation to be separated. These measurements are proposed and have not been performed.

Parallel viable-count assays, zinc-release profiles, corrected ROS measurements, and bacterial adhesion measurements would help relate any structural change to activity. Comparing direct contact with particle-free leachate and contact-separated exposure at matched measured dissolved-zinc concentrations would help test whether RNC acts mainly through release or contact. A factorial analysis including RNC and ZnO factors is needed before claiming a positive interaction; a stronger effect than one comparator alone does not establish synergy.

### 2.8. Particle Characterization and Network Design Limitations

TEM, DLS, zeta-potential, and mechanical measurements were not performed. TEM of the isolated ZnONPs and RNC is needed to establish morphology and particle-size distributions. DLS and zeta-potential measurements should be performed for the separate dispersions and relevant mixtures at a defined pH, ionic strength, concentration, and temperature. DLS gives an apparent equivalent-sphere hydrodynamic size for anisotropic particles and cannot replace direct measurement of cellulose length and width. Compression testing of equilibrated hydrated beads and matched SA/PVA, SA/PVA/RNC, and SA/PVA/ZnONPs controls should quantify deformation resistance and recovery. Cyclic compression before and after reuse would assess mechanical durability. Bead geometry, loading rate, and the model used to infer mechanical parameters should be reported.

RNC was maintained at the nominal loading of 1 wt%, and the preparation used a 10 wt% CaCl_2_ bath and a 20 min holding time. These were fixed preparation conditions, not experimentally determined optima. Consequently, the independent and interacting effects of RNC loading, cross-linker concentration and cross-linking time remain unresolved. Future work should include an RNC-free control and graded RNC loadings, together with graded CaCl_2_ concentrations and holding times. PVA and ZnO contents, droplet size, bath-to-feed ratio, mixing, washing, and hydration conditions should be controlled. A factorial design would help resolve interactions. Responses should include gel yield or insoluble fraction, swelling kinetics, mass loss, hydrated-state compression and rheology, zinc release, and antibacterial activity. Cross-link density should not be inferred from swelling or SEM alone.

### 2.9. Characterization After Reuse and pH Conditioning

Fresh and reused specimens should be compared after selected cycles using standardized imaging, dry-mass recovery, hydrated-state mechanical testing and quantitative residual-zinc analysis after complete digestion. SEM-EDS can map elemental distribution but cannot replace bulk zinc quantification by ICP-OES or ICP-MS. A zinc mass balance should include the initial hydrogel, recovered hydrogel, antibacterial media, wash solutions, and detached material. Rinsing-and-drying controls without bacteria would help separate processing damage from the effects associated with antibacterial exposure. This analysis is needed to determine whether declining activity tracks zinc depletion, network damage, or fouling.

For pH conditioning, time-resolved zinc release, residual zinc, calcium loss, swelling, mass loss, and morphology should be compared under the same pH, duration, and temperature used in the activity assay, with initial and final pH recorded. Following conditioning, a standardized rinse and antibacterial assay in a common medium would distinguish material alteration from direct pH effects on bacterial growth. Separate controls are needed if antibacterial performance is also tested directly at each pH. Acid-enhanced zinc release during conditioning may coexist with lower subsequent activity if zinc is lost before the antibacterial assay. This proposed explanation requires a measured release and residual-content balance.

PVA is largely nonionic over the tested range, so its anticipated influence on pH response is indirect through hydration, chain association and transport rather than a major intrinsic acid–base transition. Increasing PVA could either support water uptake or constrain expansion, depending on network organization. ZnO can dissolve more readily under acidic conditions; the resulting loss or redistribution of zinc species could alter the network and subsequent antibacterial response. The 1.0 wt% ZnO selection does not establish an optimum for pH stability, and the present data do not resolve PVA-by-ZnO-by-pH interactions. A factorial study with fixed buffer composition or documented ionic strength, pH before and after exposure, swelling, calcium/zinc release, and residual mass would be needed to distinguish these effects.

### 2.10. Safety Limitations and Application Prospects

No mammalian-cell cytotoxicity, hemocompatibility, irritation, or food-contact migration tests were performed. Biocompatibility cannot be inferred from the individual polymers, plant-assisted synthesis, or antibacterial activity. The finished composite and its released species require evaluation for the intended contact route and exposure [64]. As future work, extract-based and direct-contact cytotoxicity testing should follow an appropriate ISO 10993-5 protocol, with extraction conditions selected under ISO 10993-12. Fibroblasts and keratinocytes would be relevant models for a wound-contact application. Concentration- and time-dependent cell viability, morphology, and membrane integrity should be assessed with positive, negative, and material-interference controls. Orthogonal endpoints are needed where ZnO or extract constituents interfere with colorimetric or fluorescent assays. The antibacterial exposure range should be compared with the cytotoxicity range to assess whether a useful efficacy–tolerability window exists.

Food-preservation use requires separate migration and exposure assessment. An EFSA evaluation of ZnO in specified polymer food-contact materials emphasized soluble-zinc migration [65]; those findings cannot be transferred directly to this water-rich composite. Future work should quantify total and dissolved zinc and investigate possible particle release into relevant food simulants under intended contact times and temperatures, including acidic conditions. Matrix-derived migrants, actual food-storage efficacy and product quality should also be assessed. A favorable cell-viability result alone would not establish food-contact safety. Accordingly, biomedical and food-preservation applications remain research prospects rather than validated uses. Antimicrobial synthetic and natural polymeric nanofibers have also been reviewed for wound dressings [66], although electrospun dressings differ from the present freeze-dried beads. Mechanistic work on ZnO-exposed Streptococcus mutans combined ROS, membrane integrity, membrane potential, and biofilm measurements [67]. Such complementary measurements illustrate a possible validation strategy, but organism, medium, and material differences prevent direct transfer of those mechanistic conclusions to these beads. Neither dissolved-zinc release kinetics nor nanoparticle leaching was measured. Repeated activity over the plotted cycles and storage interval is a functional observation, not direct evidence of long-term structural stability. Time-resolved release, residual zinc, mass recovery, and mechanical properties during hydrated aging under the intended exposure conditions are required before sustained-release, nonleaching or durable-use claims can be made. Food-related and biomedical applications are retained only as prospects for subsequent validation.

## 3. Materials and Methods

### 3.1. Materials and Reagents

Sodium alginate (SA), zinc sulfate (ZnSO_4_, ≥99.0%), polyvinyl alcohol (PVA), and sodium hydroxide (NaOH, ≥96.0%) were purchased from Sinopharm Chemical Reagent Co., Ltd. (Shanghai, China). *Escherichia coli*, *Pseudomonas aeruginosa*, and *Staphylococcus aureus* were provided by the School of Pharmacy, Changzhou University. All chemical reagents were of analytical grade and used without further purification. Deionized water was used throughout all experiments.

### 3.2. Preparation of SA/PVA/RNC/ZnONPs Composite Hydrogel

Preparation of ZnONPs: Zinc oxide nanoparticles (ZnONPs) were synthesized using dandelion extract. Reference [19] describes saffron-assisted ZnO synthesis and is cited only as plant-assisted synthesis context; it does not verify this dandelion protocol. The brief procedure is as follows: first, dandelion was pulverized with a grinder, and 10 g of the powder was immersed in 100 mL of distilled water, followed by stirring extraction in a 60 °C water bath for 20 min. After filtration through filter paper, the dandelion extract was obtained for subsequent use 10 mL of the above extract was thoroughly mixed with 100 mL of zinc sulfate solution, and 2 M NaOH solution was added dropwise under stirring. The mixture was further stirred at 60 °C for 1 h to allow the reaction to proceed, during which a milky white precipitate gradually formed in the system. Upon completion of the reaction, the precipitate was collected by centrifugation (8000 rpm, 5 min). The obtained solid was washed repeatedly with ethanol and distilled water 2–3 times in sequence to sufficiently remove impurities, followed by drying. The dried powder was then calcined in a muffle furnace at 400 °C for 2 h to obtain zinc oxide nanoparticles (ZnONPs).

Preparation Method of Rape Straw Nanocellulose (RNC): Ren et al. [20] review agricultural-waste nanocellulose preparation and applications; that review provides background rather than a verified source for every condition in the procedure below. First, the raw rape straw was thoroughly washed with deionized water to remove surface impurities (e.g., soil, dust and soluble substances), and then dried in an oven at 60 °C until a constant weight was achieved to eliminate residual moisture. Subsequently, the dried rape straw was crushed into fine particles using a high-speed grinder, and the obtained powder was sieved through a 20–80 mesh sieve to ensure uniform particle size distribution, which was then stored in a desiccator for subsequent use. For the chemical pretreatment, 4 g of the sieved RS powder was accurately weighed and added into 50 mL of the prepared treatment solution, which was composed of 7 g NaOH, 7 g ultra-high pressure (UHP), and 1 mL glycerol polyether (GPE) per 50 mL of deionized water. The mixture was transferred into a three-necked flask and reacted at 110 °C under constant magnetic stirring for 60 min to fully remove hemicellulose, lignin and other non-cellulosic components in the rape straw. After the reaction was completed, the treated RS sample was collected by vacuum filtration and repeatedly rinsed with deionized water until the pH value of the washing filtrate reached neutral (pH = 6.8–7.2), so as to completely remove the residual treatment reagents. The rinsed sample was then dried in an oven at 70 °C until constant weight. Finally, the dried sample was subjected to continuous grinding in a high-speed planetary ball mill for 2 consecutive days under optimized parameters (rotating speed: 400 r/min, ball-to-material ratio: 10:1), and the rape-straw-derived cellulose material (RNC) was obtained; its nanoscale dimensions and particle morphology remain to be verified.

Preparation of SA/PVA/RNC/ZnONPs composite hydrogel: The as-prepared rape-straw-derived cellulose material (RNC, 1 wt%) dispersion was mixed with sodium alginate (SA, 1 wt%), polyvinyl alcohol (PVA, 0.4 wt%), and zinc oxide nanoparticles (ZnONPs, 0.5 wt%) in proportion, and continuously stirred in a 50 °C water bath to achieve sufficient dispersion of each component. Subsequently, the prepared SA/PVA/RNC/ZnONPs solution was loaded into a syringe and added dropwise at a constant rate into 10 wt% calcium chloride solution, forming gel beads instantaneously. After the dropwise addition, the system was allowed to stand for 20 min for cross-linking under the selected preparation conditions. The gel beads were collected, pre-frozen at −80 °C for a period of time, and then freeze-dried to finally obtain SA/PVA/RNC/ZnONPs composite hydrogel beads. This paragraph reports a nominal preparation using 0.4 wt% PVA and 0.5 wt% ZnONPs, whereas the screening sections select 0.8 wt% PVA for swelling and 1.0 wt% ZnONPs for antibacterial activity. These are distinct compositions and must not be treated as interchangeable. The original formulation records are required to establish which composition was used in each characterization and performance assay.

**Figure 10:**
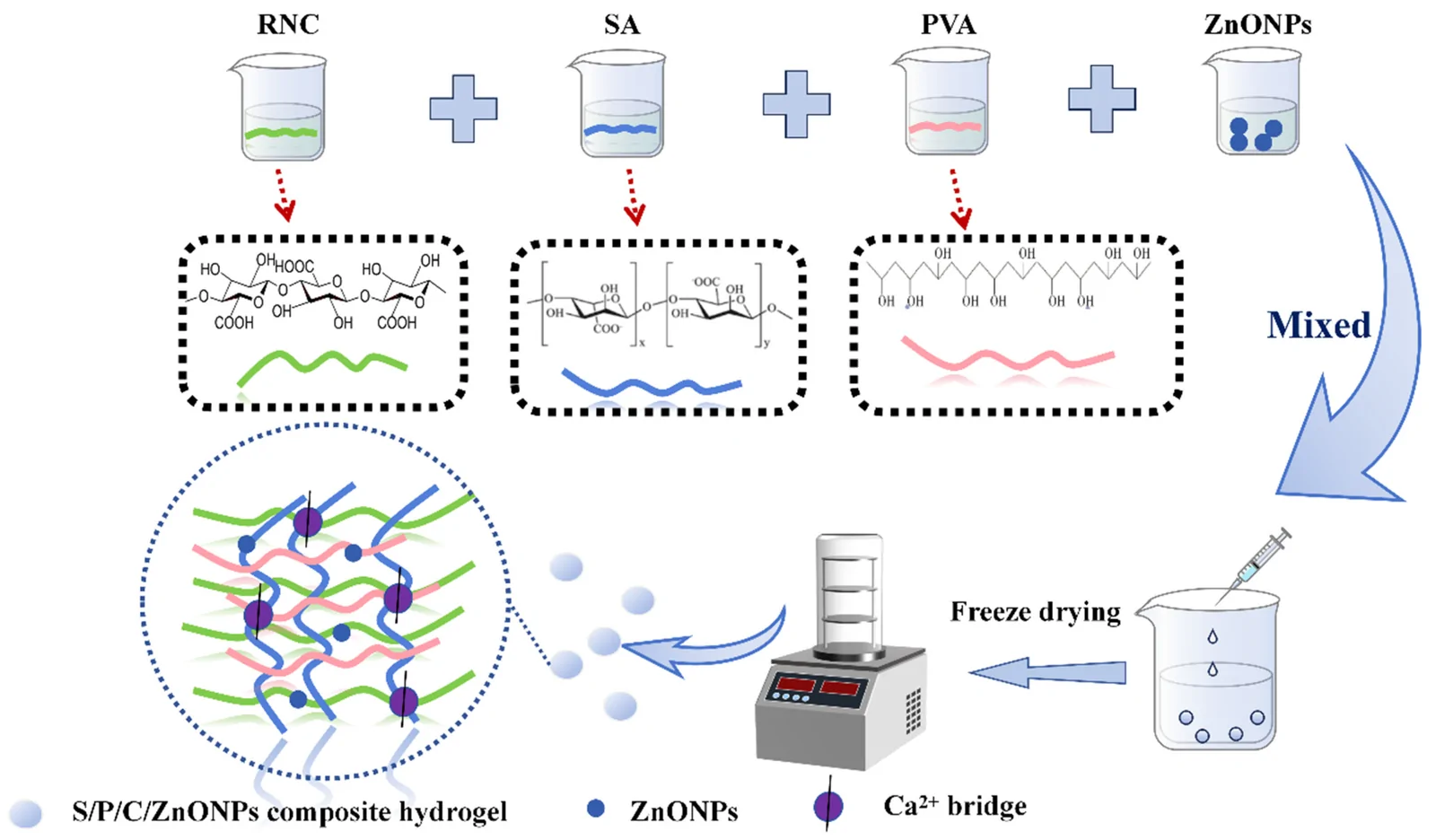
Preparation of SA/PVA/RNC/ZnONPs composite hydrogel.

The preparation process is illustrated in Figure 10.

### 3.3. Characterization of SA/PVA/RNC/ZnONPs Composite Gel Beads

#### 3.3.1. Scanning Electron Microscopy (SEM)

The surface microstructure and cross-sectional morphology of the composite material and other control materials were observed using a scanning electron microscope (Supra55, Zeiss, Germany).

#### 3.3.2. Fourier Transform Infrared Spectroscopy (FTIR)

Fourier transform infrared spectroscopy (Nicolet iS50, Thermo Fisher, Waltham, MA, USA) was employed to characterize the chemical composition and functional group information of the composite material and other control materials. Spectra were collected over the range of 4000–500 cm^−1^, and the interactions between various components were analyzed based on the position and intensity of characteristic absorption peaks.

#### 3.3.3. X-Ray Diffraction (XRD)

X-ray diffraction (XRD, D/max 2500 PC, Tokyo, Japan) experiments were performed on the composite material and other control materials using an X-ray diffractometer with Cu Kα radiation (λ = 0.154 nm). The diffraction angle 2θ ranged from 5° to 80°.

### 3.4. Effect of PVA Content on Swelling Performance and Water Loss Rate of Aerogels

#### 3.4.1. Effect of PVA Content on Swelling Performance of Aerogels

To investigate the influence of poly(vinyl alcohol) content on the swelling behavior of composite gels, a series of gel samples with different PVA mass fractions (0.2, 0.4, 0.6, 0.8, 1.0, and 1.2 wt%) were prepared, and the dry weight of each sample was controlled at 0.1 g. The as-prepared gels were completely immersed in deionized water, taken out every 30 min, and weighed after gently wiping off excess surface water with filter paper. The mass changes at each time interval were recorded. The swelling ratio of the samples was calculated according to Equation (1).

The swelling behavior of gel materials in different pH environments is an important consideration in their practical applications. The gel sample with the optimal swelling performance was selected, weighed 100 mg, and immersed in solutions of different pH values. It was taken out every 30 min, weighed after drying the surface moisture, and the data were recorded to evaluate the effect of pH on the swelling behavior of the gel. The swelling ratio was calculated according to Equation (1):

(1) $$Swelling\;ratio\left(\%\right)=\frac{W_{1}-W_{0}}{W_{0}}\times 100$$

where *W*_1_ is the mass of the aerogel after water immersion, mg, and *W*_0_ is the initial mass of the aerogel, mg.

#### 3.4.2. Effect of PVA Content on Water Loss Rate of Aerogels

Gel samples with different PVA contents (from 0.2 wt% to 1.2 wt%) were placed in a constant-temperature drying oven at 60 °C for dehydration treatment. The samples were taken out sequentially at preset time intervals (0.5, 1, 2, 3, 4, 5, 10, 15, 20, and 25 min), and the mass changes were recorded after weighing. The water loss rate was calculated according to Equation (2) to investigate the effect of poly(vinyl alcohol) content on the water retention performance of the gels. The calculation formula (Equation (2)) is as follows:

(2) $$Water\;loss\;rate\left(\%\right)=\frac{M_{0}-M_{1}}{M_{0}}\times 100$$

where *M*_0_ is the mass of the aerogel after water immersion, mg; *M*_1_ is the mass of the aerogel after drying, mg.

### 3.5. Antibacterial Activity Test

#### 3.5.1. Effect of Composite Gels on Antibacterial Performance

The agar well diffusion method was employed to evaluate the antibacterial activity of the composite gels against *Escherichia coli*, *Pseudomonas aeruginosa*, and *Staphylococcus aureus*. The brief procedure is as follows: 100 μL of bacterial suspension (10^6^ CFU/mL) was uniformly spread on the surface of LB solid medium; round wells with a diameter of 9 mm were punched on the medium using a hole puncher; 0.025 g of dried gel samples were accurately weighed and filled into the wells, respectively. After incubation at 37 °C for 12–16 h, the diameters of the inhibition zones were measured.

#### 3.5.2. Effect of ZnONPs Dosage on Antibacterial Performance

This study investigated the antibacterial effect of zinc oxide nanoparticles (ZnONPs) at different contents in the gel system. The agar diffusion method was used to evaluate the antibacterial activity of SA/PVA/RNC/ZnONPs gels against Escherichia coli, Pseudomonas aeruginosa, and Staphylococcus aureus, to select a nominal ZnONPs loading within the tested range. The mass fractions of ZnONPs were set as 0.05, 0.1, 0.2, 0.5, 1.0, and 1.5 wt%, respectively.

#### 3.5.3. Effect of Composite Gel Dosage and Incubation Time on Antibacterial Performance

In this study, *E. coli*, *S. aureus*, and *P. aeruginosa* were selected as test strains to optimize the antibacterial dosage and time. At the initial stage of the experiment, the initial concentration of bacterial suspension was adjusted to 10^8^ CFU/mL using the plate counting method. Gels containing antibacterial components at different dosages of 0.2, 0.4, 0.6, 0.8, and 1.0 g/L (0.4, 0.8, 1.2, 1.6, and 2.0 g/L) were prepared separately, and the mixtures were incubated under shaking at 37 °C. Samples were collected at set time intervals; 0.1 mL of bacterial suspension was accurately pipetted each time, serially diluted, and spread on LB agar plates. After incubation at 37 °C for 12–16 h, the surviving colonies were quantitatively analyzed by colony counting method, and the antibacterial rate was determined.

#### 3.5.4. Reusability and Stability of Composite Aerogels

Reusability serves as a critical evaluation criterion for assessing the practical application potential of composite gels. To investigate the cyclic antibacterial performance of the material in aqueous environments, *E. coli*, *S. aureus*, and *P. aeruginosa* were selected as the test strains in this study. Specifically, 0.1 g of the dried SA/RNC/PVA/ZnONPs composite gel was accurately weighed and added to 50 mL of bacterial suspension with a concentration of 10^8^ CFU/mL. After incubation at a constant temperature for a designated period, the number of viable bacteria in the system was determined. Following each antibacterial cycle, the gel was rinsed and dried before being reused in the next run. Over eight consecutive antibacterial test cycles, the variation in the inhibitory efficacy of the gel against the three bacterial strains was systematically recorded and analyzed.

Stability constitutes an essential performance foundation for composite gels to be practically applied as antibacterial materials. In this study, the as-prepared SA/RNC/PVA/ZnONPs gel was stored at room temperature, and its antibacterial performance was examined at various storage time points, with the experimental results recorded and analyzed to evaluate the stability of the material during long-term storage. Meanwhile, maintaining stable antibacterial efficacy in complex aqueous environments represents a key criterion for promoting the large-scale application of such materials. Accordingly, the gel was immersed overnight in solutions with pH values ranging from 1 to 9, respectively. After thorough rinsing with deionized water, antibacterial tests were conducted, and the differences in antibacterial performance under diverse acid–base conditions were recorded and compared, thereby assessing the acid–base resistance stability of the material.

### 3.6. Statistical Reporting and Proposed Analysis

The supplied experimental description does not document independent replicate numbers, error-bar definitions, statistical software, or inferential tests for Figure 5 and Figure 6. Consequently, comparisons are presented descriptively and no *p*-values are asserted. The analysis below is prospective and must not be reported as completed until the original records and replicate data have been verified.

For a prospective comparison, independently prepared batches should be the experimental units; multiple wells from one batch are technical replicates and should not inflate n. For each bacterial species, one-way ANOVA (V28.0) with Tukey-adjusted pairwise comparisons is appropriate for independent formulation groups if residual and variance assumptions are supported. Welch ANOVA with Games–Howell comparisons can address unequal variances; Kruskal–Wallis with multiplicity-adjusted Dunn comparisons may be considered when a rank-based analysis is appropriate. Where treatments share preparation batches, include batch as a blocking or random effect rather than assuming independence. Report the selected test, assumption checks, software and version actually used, independent n, individual values, mean and SD or justified alternatives, effect sizes and confidence intervals, and multiplicity-adjusted *p*-values using a prespecified threshold such as 0.05. These are analysis recommendations, not completed statistical results.

## 4. Conclusions

SA/PVA/RNC/ZnONPs composite gel beads were prepared by ionic cross-linking and freeze-drying. Structural characterization and antibacterial assays provide an initial assessment of the formulations. Within the tested ranges, 0.8 wt% PVA was selected on the basis of swelling and 1.0 wt% ZnONPs on the basis of antibacterial screening. Antibacterial function was retained during repeated use and storage, with reduced activity after strong-acid preconditioning. These findings do not establish particle immobilization, mechanical durability, sustained zinc release, or a specific killing mechanism. Because RNC loading and cross-linking conditions were fixed, network optimization remains incomplete. Particle characterization, hydrated-state compression testing, release and mechanistic assays, and characterization after reuse and pH conditioning are needed. Cytocompatibility and food-contact migration have not been assessed; biomedical and food-preservation uses remain prospective and require application-specific validation.

## Data Availability

The original contributions presented in this study are included in the article. Further inquiries can be directed to the corresponding author.

## Abbreviations

AMR, antimicrobial resistance; SA, sodium alginate; PVA, poly(vinyl alcohol); RNC, rape-straw-derived cellulose material (the study sample label; nanoscale dimensions remain unverified); ZnONPs, zinc oxide particles referred to by the original sample label (primary-particle dimensions remain unverified); S/P, SA/PVA; S/P/C, SA/PVA/RNC; S/P/ZnONPs, SA/PVA/ZnONPs; S/P/C/ZnONPs, SA/PVA/RNC/ZnONPs. C in the figure shorthand denotes the cellulose component. SEM, scanning electron microscopy; TEM, transmission electron microscopy; DLS, dynamic light scattering; FTIR, Fourier transform infrared spectroscopy; XRD, X-ray diffraction; EDS, energy-dispersive X-ray spectroscopy; ICP-OES, inductively coupled plasma optical emission spectrometry; ICP-MS, inductively coupled plasma mass spectrometry; EPR, electron paramagnetic resonance; ROS, reactive oxygen species. RS, rape straw; GPE, glycerol polyether; LB, Luria–Bertani medium; CFU, colony-forming units; ISO, International Organization for Standardization; EFSA, European Food Safety Authority; wt%, weight percent.
